# Rare appearance of an odontogenic myxoma in cone-beam computed tomography: a case report

**DOI:** 10.15171/joddd.2016.010

**Published:** 2016-03-16

**Authors:** Arash Dabbaghi, Nafiseh Nikkerdar, Soheyla Bayati, Amin Golshah

**Affiliations:** ^1^Assistant Professor, Department of Oral and Maxillofacial Radiology, School of Dentistry, Jundishapur University of Medical Sciences, Ahvaz, Iran; ^2^Assistant Professor, Department of Oral and Maxillofacial Radiology, School of Dentistry, Kermanshah University of Medical Sciences, Kermanshah, Iran; ^3^Assistant Professor, Department of Orthodontics, School of Dentistry, Kermanshah University of Medical Sciences, Kermanshah, Iran

**Keywords:** Cone-beam computed tomography, odontogenic tumor, myxoma

## Abstract

Odontogenic myxoma (OM) is an infiltrative benign bone tumor that occurs almost exclusively in the facial skeleton. The radiographic characteristics of odontogenic myxoma may produce several patterns, making diagnosis difficult. Cone-beam computed tomography (CBCT) may prove extremely useful in clarifying the intraosseous extent of the tumor and its effects on surrounding structures. Here, we report a case of odontogenic myxoma of the mandible in a 27-year-old female. The patient exhibited a slight swelling in the left mandible. Surgical resection was performed. No recurrence was noted. In the CBCT sections, we observed perforation of the cortical plate and radiopaque line that extended from the periosteum, resembling "sunray" appearance—a rare feature of OM—which could not be assessed by panoramic radiography.

## Introduction

 Odontogenic myxoma (OM) is a relatively rare tumor of mesenchymal origin.^1,4-7^ It represents 3-6% of all the odontogenic tumors.^1^ Myxoma is a benign, intraosseous, locally invasive and non-metastasizing neoplasm of the jaw bone. The posterior region of the jaw is the most frequent area where the neoplasia occurs.^1^ Pain, dysesthesia, ulceration, soft tissue invasion and tooth mobility are the symptoms of patients, although the majority of them have no clinical signs or symptoms.^3^ Radiographically, OM exhibits several patterns such as unilocular, multilocular, pericoronal and radiolucent/radiopaque patterns.^2^ Most reported OMs are multilocular with coarse or angular trabeculations.^8^ The aim of this report is to present a case of OM in the mandibular left molar/ramus region of a 27-year-old female that had a rare CBCT appearance with periosteal reaction.

## Case report

 A 27-year-old female patient who attended a dental clinic in Dezful presented for a serious 3-year complaint of a slight swelling in the left mandibular region, which had developed over a course of one year.

 The patient initially underwent periodontal treatment for the involved teeth, which included extraction of the third molar in the same involved side. After 6 months, the patient returned to the clinic, reporting no improvement but progression of the disease, with slight facial asymmetry and numbness on the left side of the lower lip. Then, she was referred to the Department of Oral and Maxillofacial Radiology of Jundishapur University of Medical Sciences, while complaining about dull, diffuse and chronic pain and feeling heaviness on the left side of the mandible. The physical examination showed expansion of both cortical plates in the left posterior mandibular alveolus. The swelling was bony hard in palpation with normal overlying mucosa and no ulceration. The medical and family history revealed nothing of note and she did not have any oral habits.

**Figure 1:**
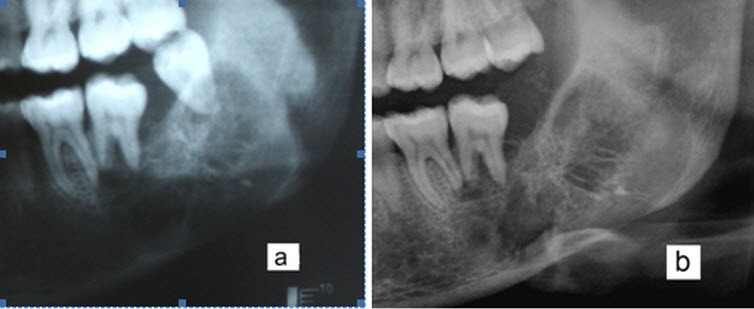
Cropped panoramic radiograph showed a mixed radiolucent/radiopaque lesion with a relatively well-defined border between the left mandibular first molar and ramus. Before extraction (a). Six months after the extraction of third molar (b). Tooth displacement and root resorption were also seen on the panoramic radiograph.

**Figure 2:**
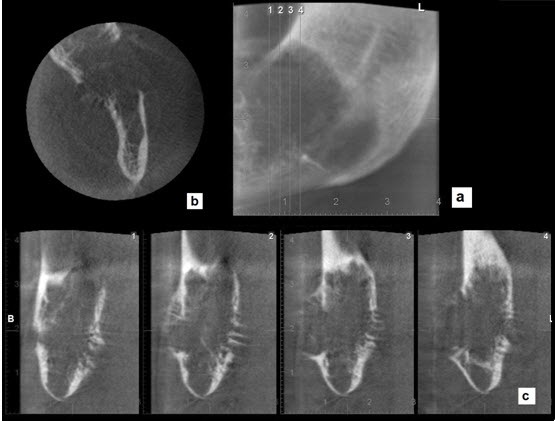
Panoramic (a) axial (b) and cross sections (c) showed perforation of the cortical plate and displayed septa at the periphery of the lesion.

**Figure 3:**
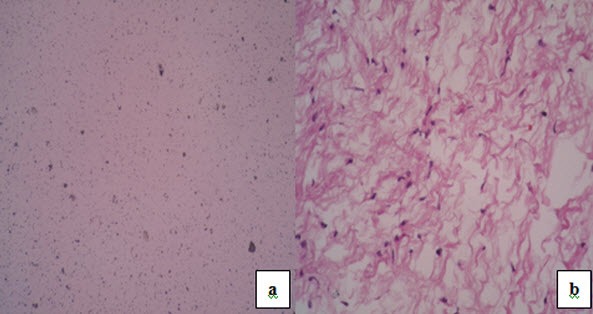
Photomicrographs; (a) low power; loose myxoid stroma contained only a few collagen fibril; (b) high power; the tumor was composed of stellate, spindle-shaped and round cells in a myxoid stroma.

 The rotational panoramic and periapical radiography showed a mixed radiolucent/radiopaque lesion with a relatively well-defined border from the first molar to the ramus. Internally, the lesion exhibited a straight and sharp trabecular pattern (Figure 1; a & b). However; a CBCT scan was ordered to obtain superior images to detect its nature and effect on the surrounding structures. The CBCT sections displayed lingual and buccal cortical plate perforation and showed septa at the periphery of the lesion, presenting as a “sunray” spicular pattern. The lesion was considered to represent an invasive lesion such as osteogenic sarcoma due to periosteal reaction and perforation of buccal and lingual plates (Figure 2; a,b and c ). From these preliminary observations, we came to the following diagnostic hypotheses: Sarcoma (especially osteogenic sarcoma), OM and ameloblastoma. The histopathological examination after incisional biopsy revealed a benign odontogenic tumor composed of haphazardly arranged stellate, spindle-shaped cells in an abundant, loose myxoid stroma that contained collagen fibers and suggestive of OM. This tumor was removed surgically by block resection with wide margins under general anesthesia. The gross pathology of these specimens showed a whitish–grey glistening or gelatinous mass with minimal true encapsulation. The excisional biopsy also confirmed the diagnosis of OM (Figure 3; a & b). Regular follow-up with image guidance was carried out to detect the recurrence of this lesion. After a two-year follow-up, no recurrence was reported.

## Discussion

 OM is slightly more common in females, with a male-to-female ratio of 1:1.5. Two-thirds of OM cases are located in the mandible and the other one-third is located in the maxilla.^9^ The posterior region of the jaw is where the neoplasia most frequently occurs.^6^ Myxomas are slow-growing and locally destructive tumors that display behavior similar to malignant tumors in some cases but do not metastasize to lymph nodes. ^10^

 Myxomas have been reported to show a wide spectrum of radiographic appearances,^11^ such as pericoronal, unilocular, multilocular and radiolucent/radiopaque patterns with an impacted tooth; thus radiographic appearance is important to establish diagnosis. ^6^ In most cases, the septa are curved and coarse, but straight and thin septa represent a tennis racket-like or stepladder-like pattern, which is a rare characteristic of OM.^1^

 Conventional radiography is usually the first imaging modality to be performed and may reveal something from small periapical radiolucencies to large multilocular lesions.^12^ Tooth displacement and root resorption are more reliably observed by conventional radiography^13^ but CBCT appears more convenient and is useful for observing the maxillofacial lesion in detail. These pieces of information include precise extension of the lesion, perforation, periosteal reaction and so on.^14^

 In this case, we observed the perforation of the cortical plate and radiopaque line that extended from periosteum, resembling a “sunray” appearance in CBCT sections, which could not be assessed by panoramic radiography. This “sunray” appearance suggested the diagnosis of an osteogenic sarcoma, but the histopathological examination after incisional biopsy was suggestive of OM.

 On occasion, expansion of a small area with straight septa may project over an intact outer bony cortex and give a speculated appearance seen in osteogenic sarcoma. Careful inspection of this expansion area reveals a thin but intact outer cortex that will not be seen in osteogenic sarcoma,^1,15^ but in our case, lingual and buccal cortical plates showed perforation and septa at the periphery of the lesion, presenting a true “sunray” spicular pattern that was not apparent on panoramic radiography. Thoma^16^ has described two types of myxoma: odontogenic myxoma and osteogenic myxoma. The former is considered as benign while the latter is viewed as malignant and as a sarcoma with similar changes.

 The “sunray” appearance of OM is rare and has been reported in few studies.^17-20^

 Some studies reported OM cases with unusual periapical cortical reaction on conventional radiography, which mimicked osteogenic malignant disease.^17-19^

 MacDonald-Jankowski et al^20^ reported lesions that displayed a “sunray” spicular pattern on CT.

 In future, the application of three-dimensional CT or CBCT and full access to all sections may reveal new information about the lesions and their impact on other structures. Furthermore, since myxoma has a high recurrence rate, CBCT can be the method of choice for the diagnosis and follow-up of OM cases.

## Acknowledgments

 The authors would like to thank Dr. Shahrokh Raeisian, Department of Oral and Maxillofacial Surgery, and Dr. Saedeh Attarbashi Moghaddam, Department of Oral and Maxillofacial Pathology, Ahvaz Jundishapur University of Medical Sciences Dental School.

## Authors’ contributions

 AD, NN, and SB performed the clinical and radiographic examinations. NN and AG drafted the manuscript. All authors contributed to critical revision of the manuscript, and have read and approved the final manuscript.

## Funding

 The authors report no funding for this article.

## Competing interests

 The authors declare that they have no competing interests with regards to authorship and/or publication of this paper.

## Ethics approval

 The authors declare that the individual, whose data were reported in this article, has given written consent to the authors and the Ethics Committee of Ahvaz Jundishapur University of Medical Sciences for the publication of this paper.
